# Bladder dysfunction depends on many variables in children with posterior urethral valves

**DOI:** 10.1590/S1677-5538.IBJU.2021.0046.1

**Published:** 2021-12-21

**Authors:** Lisieux Eyer de Jesus

**Affiliations:** 1 Hospital Federal dos Servidores do Estado Rio de Janeiro RJ Brasil Hospital Federal dos Servidores do Estado, Rio de Janeiro, RJ, Brasil; 2 Universidade Federal Fluminense Hospital Universitário Antônio Pedro Niteroi RJ Brasil Hospital Universitário Antônio Pedro, Universidade Federal Fluminense - UFF, Niteroi, RJ, Brasil

## COMMENT

We congratulate the authors for a very good paper on an important topic about what the Pediatric Urologists community still has many unanswered questions.

The paper compares neonates (6-18 days) to young children (3-19 months), and conclusions should apply only to these age groups: late diagnosis in older children may present other characteristics (1). The dynamic nature of the problem implies that valve bladders differ according to age, so that overactivity predominates in the first 5 years of life, while detrusor failure typically appears in late school-aged children or adolescents, complicating the analysis of bladder functions in papers that adopt follow-up periods including different ages. Age also affects the epidemiology of kidney failure (especially end-stage failure), that predominates in the first years of life and adolescence/young adulthood.

The only statistically significant finding of the paper (higher % PVR in the late treatment group) remains to be proved as clinically meaningful, as the differences between the two groups are small. An important secondary result is the confirmation that definitive harming to renal function relates to late diagnosis, even when late diagnosis is defined as diagnosis in the first two years of life. Unfortunately, diagnostic delay of posterior urethral valves remains common in low-medium income countries, relating mainly to inappropriate prenatal care.

Bladder dysfunction depends on many variables, so that defining the factors that modulate this problem is difficult in practice. Different patients may show varying degrees of bladder dysfunction depending on the degree of obstruction (posterior urethral valves may vary anatomically from short sub-occlusive valves to almost complete urethral occlusion), polyuria, severity of secondary vesicoureteral reflux (2), and the detrusor response to compensate high voiding pressures. Interestingly, tissue response for aggressions and tissue remodeling differ between fetuses, neonates/children younger than 3 months, and older pediatric patients/adults. Profiles of proteoglycans, collagens, growth factors, and inflammatory reaction differ from adults. Linear incisions usually heal without scarring in fetuses. Collagen deposition in fetal wounds is quick, highly organized and the proportion of collagen types differs from older individuals (3). Perinatal treatment histological results may differ from those corresponding to treatment after the first trimester of life. Despite most research referring to skin mechanisms of scarring, the process of bladder remodeling may differ between very young children and older ones, which may explain differing results concerning bladder function whenever the relief of urethral obstruction occurs beyond the very first months of life.

The usage of desmopressin to treat posterior urethral valves patients remains controversial, as polyuria in these patients derives from definitive harm to the distal tubules (4). Perhaps, though, this is also modulated by timing of the treatment, and we can presume worse or irreversible tubular harming in protracted cases. Also, in my experience presentation of posterior urethral valves as urinary retention is quite rare, especially among babies and toddlers. I wonder if some of the cases presented in this research disguise urinary tract infections. It would be reasonable to assume that the data available would convince most groups to follow this strategy but the best moment to apply these measures has been subject to controversy.

The authors suggest performing a first urodynamic study within 6 weeks after closure of the neural tube defect, looking for a subgroup of patients with greater risk due to reduced bladder capacity and compliance and high detrusor leak point pressure. Urodynamic studies in children are challenging and it is of utmost importance to know what to look for.

The authors went beyond, analyzing the updated scientific evidence on CIC indications, how to do it, how to train parents and which catheters to use in different scenarios, from diameter to hydrophilic coatings. Pharmacological therapies have also been addressed. A long list of studies are presented showing indications and results with the role of anticholinergics, beta3 agonists, alfa-blockers and botulinum toxin.

This is a must-read paper for those dealing with children with neurogenic bladder.
